# Legal Notes for Institutional Workers

**Published:** 1919-10-04

**Authors:** 


### LEGAL NOTES FOR INSTITUTIONAL WORKERS.

### Professional Secrecy.

Once again the different points from which medical men and lawyers view some of the incidents in which both professions are interested have led to divergence of opinion. Sir John Tweedy, in his capacity of President of the Medical Defence Union, felt it incumbent to write to the *Lancet* protesting against certain utterances of Mr. Justice Bray in a recent trial at the Worcester Assizes. The facts were that a practitioner was summoned to the assistance of a woman who had obviously been quite recently delivered of a child. No child was anywhere to be seen, and all knowledge of its whereabouts was denied. The doctor devoted his efforts towards saving the life of his patient, who is described as having been in a critical condition; and in this he was successful. He did not immediately notify the police of his suspicion that crime might have been committed, or might be about to be committed, because he felt that he had gained knowledge of the child's birth in professional confidence. For this view he was called up after the trial and lectured by the judge, who told him that the law recognises no such excuse for failure to comply with the ordinary duties of citizenship. The law recognises "professional confidence" only for its own practitioners; neither doctors nor priests may regard it as justification for failure to promote the ends of justice. While this is so, it is yet extremely doubtful whether any jury could be found to convict either priest or medical man for failure to disclose information acquired professionally. Sir John Tweedy and the Defence Union, who are practically encouraging doctors to break the letter of the law in this respect, are therefore in a strong position—made stronger in this case by the weakness of the argument as propounded from the Bench.

### A Certifying Surgeon's Slip.

A case of special interest to certifying surgeons is *Chippendale* v. *High Explosives, Ltd.* (146 L. T. J. 21). The applicant for compensation was a workman in the employment of the respondents, and he alleged that he was suffering from an industrial disease specified in the amended third schedule to the Workmen's Compensation Act, 1906. He obtained a certificate from the certifying surgeon under the Factory Acts that he was suffering from poisoning by trinitrotoluene ("T.N.T."). It was not contradicted that the certificate which was put in in support of the applicant's case was to the effect that he was suffering from T.N.T. poisoning. The respondents, however, contended that that particular industrial disease could not have been contracted in their works since they did not manufacture T.N.T. It transpired that there was a slip on the part of the certifying surgeon and that what he ought to have certified was that the applicant was suffering from nitrous fumes or their sequelæ. It was decided by the learned County Court judge that the poisoning was caused by nitrous fumes, and accordingly His Honour made his award in favour of the respondents. The applicant appealed. It was held that the burden was upon the applicant to show that he had contracted the disease of T.N.T. poisoning, or that the disease was due to the nature of the employment in which he was engaged; and that upon the evidence the applicant had failed to show that he had contracted T.N.T. poisoning from any employment with the respondents in which he was engaged. But held that there was no reason why, upon a proper certificate being obtained, the applicant should not commence proceedings to establish his claim to compensation in respect of the industrial disease from which he was really suffering.

